# More Than a Metabolic Enzyme: MTHFD2 as a Novel Target for Anticancer Therapy?

**DOI:** 10.3389/fonc.2020.00658

**Published:** 2020-04-28

**Authors:** Zhiyuan Zhu, Gilberto Ka Kit Leung

**Affiliations:** Department of Surgery, Li Ka Shing Faculty of Medicine, The University of Hong Kong, Pokfulam, Hong Kong

**Keywords:** cancer metabolism, one carbon metabolism, metabolic enzyme, oncogenicity, epigenetic modification

## Abstract

The bifunctional methylenetetrahydrofolate dehydrogenase/cyclohydrolase (MTHFD2) is a mitochondrial one-carbon folate metabolic enzyme whose role in cancer was not known until recently. MTHFD2 is highly expressed in embryos and a wide range of tumors but has low or absent expression in most adult differentiated tissues. Elevated MTHFD2 expression is associated with poor prognosis in both hematological and solid malignancy. Its depletion leads to suppression of multiple malignant phenotypes including proliferation, invasion, migration, and induction of cancer cell death. The non-metabolic functions of this enzyme, especially in cancers, have thus generated considerable research interests. This review summarizes current knowledge on both the metabolic functions and non-enzymatic roles of MTHFD2. Its expression, potential functions, and regulatory mechanism in cancers are highlighted. The development of MTHFD2 inhibitors and their implications in pre-clinical models are also discussed.

## Introduction

MTHFD2 (350 amino acids, 37kDa) is one of the major enzymes involved in mitochondrial folate one-carbon metabolism and is also known as NMDMC (NAD-dependent mitochondrial methylenetetrahydrofolate dehydrogenase-cyclohydrolase). Despite its well-known bifunctional dehydrogenase and cyclohydrolase activities, MTHFD2 has been reported to be required for cancer proliferation and may have profound role in tumor development and progression. This metabolic enzyme has attracted particular interests in cancer research for several reasons. Firstly, MTHFD2 is upregulated in various cancers, transformed cells, and developing embryos, but has low or undetectable level in most differentiated normal adult tissues (1). Secondly, highly expressed MTHFD2 is associated with poor disease outcomes in breast cancer (2), colorectal cancer (CRC) (3), renal cell carcinoma (RCC) (4), and hepatocellular carcinoma (HCC) (5); upregulation of MTHFD2 may also contribute to an increased risk of bladder cancer (6). Thirdly, depletion of MTHFD2 may impair aggressive phenotypes and cause cell death in multiple cancers (1). Taken together, MTHFD2 is oncogenic in nature and may serve as a prognostic indicator as well as a therapeutic target in cancers.

Yet, the physiological role of MTHFD2 in malignancy and the mechanisms contributing to its pro-oncogenic activities have not yet been fully elucidated. A better understanding of both the enzymatic and non-enzymatic functional roles of MTHFD2 is essential for the optimal targeting of this novel candidate in cancer therapy. This review aims to highlight the potential functions of MTHFD2 in cancers, particularly focusing on its diagnostic/prognostic value and the effects of its knockdown on aggressive phenotypes. We will summarize the regulatory mechanisms of MTHFD2 and the effects after its depletion, including cell morphological changes, oxidative homeostasis, and metabolite profile alterations. The non-enzymatic “moonlighting” function of MTHFD2 and the development of MTHFD2 inhibitors will also be discussed.

## MTHFD2

MTHFD2 is a bifunctional NMDMC. Its activity in transformed and non-differentiated cells was firstly detected in 1985 (7) and the cDNA cloning human MTHFD2 was isolated in 1989 (8). MTHFD2 had been reported to participate in the production of formyltetrahydrofolate for the synthesis of formylmethionyl transfer RNA required for the initiation of protein synthesis (9). The MTHFD2 protein had long been thought to be located exclusively in mitochondria until recently when it was also found to be present within the nucleus at the site of newly synthesized DNA (10).

**Figure 1:**
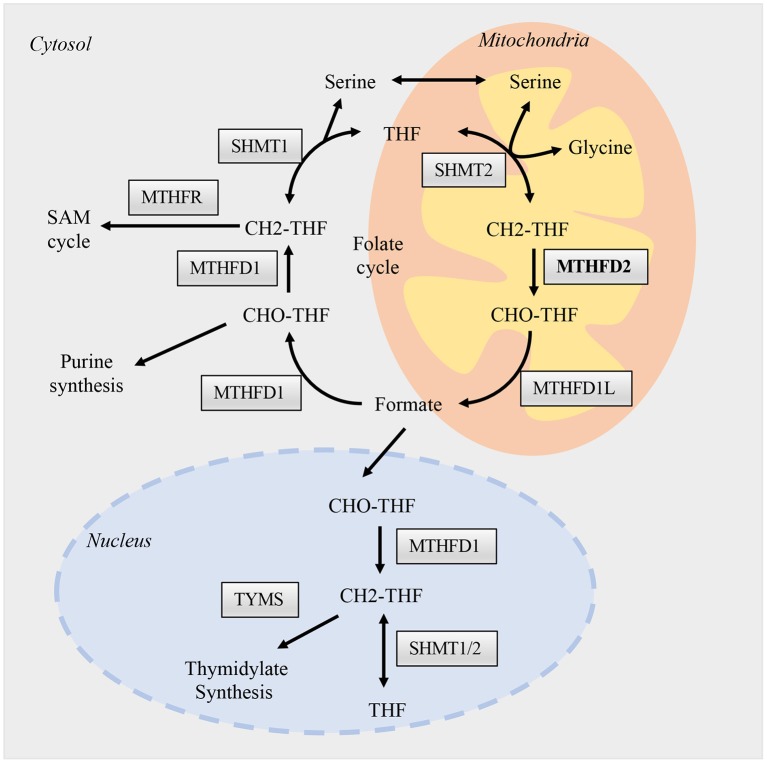
Folate one-carbon metabolism. One carbon metabolism enzymes are presented and activated in three different compartments, the nucleus, cytosol, and mitochondria. The flow of permeable metabolites linked the reactions between these compartments, such as formate, serine, and glycine. Briefly, different forms of THFs, function as carriers, transfer one-carbon units as from serine to formate in the mitochondria. Formate then supply the biosynthesis of purine in the cytosol and thymidylate in the nucleus. The reactions in mitochondria are catalyzed mainly by SHMT2, MTHFD2, and MTHFD1L; while in cytosol and nucleus are by SHMT1/2 and MTHFD1. The enzymatic functions of MTHFD2 in the mitochondria are well-studied, while its role in the nucleus is largely unknown and may hold various non-metabolic functions.

The canonical role of MTHFD2 is central to folate-mediated one-carbon metabolism in mitochondria. A one-carbon unit (1C) from serine is transferred to tetrahydrofolate (THF) by serine hydroxymethyl transferases (SHMTs) to form 5,10-methylenetetrahydrofolate (methylene-THF/CH2-THF). The 1C unit is then transferred among different forms of THFs, thus enabling the folate cycle (Figure 1). This biochemical network comprises two parallel metabolic reactions that take place in the cytoplasmic and mitochondrial compartments. In the cytoplasm, a single trifunctional enzyme named MTHFD1 comprises all the three domains (methylenetetrahydrofolate dehydrogenase, cyclohydrolase, and formyltetrahydrofolate synthetase domains), and serves as the primary functional enzyme that interconverts CH2-THF to 10-formyl-tetrahydrofolate (10-formyl-THF/10-CHO-THF). In the mitochondria, the reactions are carried out by two MTHFD isozymes, MTHFD2 and MTHFD2L (11). They catalyze the production of 10-CHO-THF via two steps. The first one is the conversion of CH2-THF to 5,10-methenyl-tetrahydrofolate (methenyl-THF/CH^+^-THF) through the dehydrogenase activity, the second step is the conversion of CH^+^-THF to 10-CHO-THF by the cyclohydrolase domain (12) (Figure 1).

MTHFD2 has been recognized to use NAD^+^ as a cofactor in the oxidation process while MTHFD2L can use both NAD^+^ and NADP^+^. A recent report, however, demonstrated that MTHFD2 can also use both NAD^+^ and NADP^+^ in rapidly proliferating cells (13), suggesting an additional uncharacterized antioxidative role. Compared with MTHFD2L isozyme, MTHFD2 was reported to have much higher expression (14) and displayed a more predominant role in maintaining mitochondrial folate pathway function as well as responding to growth factor stimulation (15). Thus, therapeutic strategies targeting the mitochondrial folate pathway could be simplified by specifically focusing on MTHFD2.

## The Role of MTHFD2 in Cancer

Although the enzymatic functions of MTHFD2 in purine synthesis have been well-studied (16), emerging evidence suggests an undefined role of MTHFD2 in embryonic development and carcinogenic transformation. MTHFD2 knockout caused embryonic deaths at 12.5 days gestation in mice (17), suggesting its indispensable role in normal embryonic development. The importance of MTHFD2 in cancers is manifested by its upregulated expression in tumor cells and the association with cancer patients outcome. Additionally, gene knockdown studies have revealed the profound impact of MTHFD2 depletion on cancers (Supplementary Table 1).

### MTHFD2 Is Overexpressed in Cancer and Predicts Prognosis

In 2014, a meta-analysis study of 19 types of human cancers showed that MTHFD2 was overexpressed in various tumors, including breast cancer, colon cancer, and liver cancer (1). This enzyme was consistently detected in transformed cells within the tumor and metastatic tissues, while having low or undetectable levels in adjacent stroma (1, 3, 18). Liu et al. (2) found that the expression level of MTHFD2 was positively correlated with clinicopathological parameters of breast cancer, such as tumor size, histological grade, and metastases. Similarly in RCC (4) and HCC (5), MTHFD2 was upregulated in tumor tissues and associated with pathological characteristics including TNM staging, diseased recurrence and patient survival. Another study of 103 pancreatic cancer patients demonstrated that highly expressed 1C metabolic enzymes (MTHFD2, ALDH1L2, or SHMT2) may predict poor Overall Survival (OS) and Disease-Free Survival (DFS) rates. Multivariate Cox proportional hazards analysis then identified MTHFD2 and ALDH1L2 expression levels as independent survival predictors for OS and DFS (19).

Findings in glioma are more controversial. Several reports, in line with the above mentioned studies, demonstrated that MTHFD2 was upregulated in glioma (20) and positively correlated with tumor grade (21). Strikingly, other groups found the reverse trend. A bioinformatics analysis study showed that MTHFD2 was among the key genes that were downregulated in glioma and that such downregulation was associated with poor prognosis (22). Another study on glioblastoma multiforme (GBM) proposed a survival-prediction gene signature in which MTHFD2 was one of the protect factors. Patients with highly expressed MTHFD2 may have a longer survival period (23). The causes and mechanisms behind the discrepant expression profiles and distinct prognostic values of MTHFD2 between glioma and other cancers remain elusive and deserve further investigations. Given the fact that GBM is particularly refractory to treatment and frequently leads to poor patient outcome, novel therapeutic targets and strategies are needed. Metabolism reprogramming is one of the most active fields in cancer research, and the effects and consequences of elevated MTHFD2 in glioma are largely unknown. Thus, experimental and clinical studies are needed to understand the diagnostic/prognostic value of this metabolic enzyme as well as its potential functional roles in glioma.

### Inhibition of MTHFD2 Causes Cancer Cell Death and Suppresses Malignant Phenotypes

RNA interference targeting MTHFD2 had been shown to suppress cancer cell malignant features and cause cell death in various cancers, including AML, CRC, HCC, RCC, glioma, breast cancer, lung cancer, ovarian cancer, and melanoma (Supplementary Table 1). The effects of MTHFD2 depletion vary among different types of cancer cells. For instance, in breast cancer, MTHFD2 knockdown suppressed cell migration and invasion. No significant effect on cell proliferation or apoptosis was observed (24). In AML cells, Pikman et al. (25) observed cell growth suppression and cell differentiation induction after depletion of MTHFD2. They further demonstrated that MTHFD2 ablation impaired leukemic establishment and progression in a human AML orthotopic xenograft model. In HCC cells, siRNA-mediated silencing of MTHFD2 inhibited cellular features associated with cancer metastasis, including cell migration, invasion, and epithelial-mesenchymal transition but no significant difference was observed on cell proliferation, apoptosis, or cell cycle distribution (5). In RCC, Lin et al. (4) described decreased cell proliferation, migration, and invasion after MTHFD2 knockdown in 786-O cells, possibly through a reduction in vimentin expression. Importantly from a therapeutic perspective, MTHFD2 down-regulation sensitized RCC cells to anti-folate chemotherapy drugs, such as methotrexate (MTX) and fluorouracil (5-FU). In CRC, MTHFD2 knockdown caused cell death under hypoxia and decreased cell growth and sphere formation ability. Furthermore, MTHFD2 suppression reduced tumor growth and significantly inhibited lung metastasis in CRC cell-derived xenograft mouse model (3, 26, 27). Targeting MTHFD2 may impair the stem-like features and chemo-resistance in lung cancer, offering an opportunity for eradicating tumors and preventing recurrence (28). In sum, MTHFD2 depletion could result in cancer cell death and impair key features associated with cancer progressions, such as proliferation, invasion, migration, and metastasis. Targeting MTHFD2 is thus a promising strategy for anti-cancer therapy.

## Regulation of MTHFD2 in Cancers

**Figure 2:**
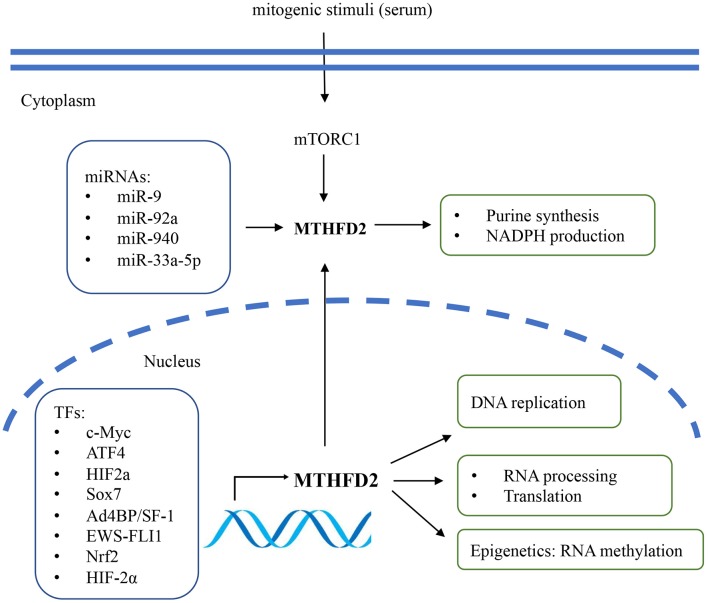
The regulatory mechanisms and biological functions of MTHFD2 in cancer. The gene expression of MTHFD2 is transcriptionally regulated by various transcriptional factors and post-transcriptionally regulated by microRNAs. Extracellular stimuli may modulate MTHFD2 expression via mTORC1/ATF4 signaling pathway. The canonical role of MTHFD2 is converting one-carbon units for *de novo* purine synthesis. It may also involve in the production of redox equivalent (NADPH) for oxidative stress defense. In the nucleus, this enzyme may play a role in DNA replication, RNA processing and translation.

Several studies have described the potential regulatory mechanisms of MTHFD2 expression, including transcriptional, post-transcriptional regulation, and extracellular stimuli (Figure 2). Various transcriptional factors (TFs) have been reported to modulate the expression level of MTHFD2. Ben-Sahra et al. (29) demonstrated that MTHFD2 expression was regulated by the mammalian target of rapamycin complex 1 (mTORC1) signaling pathway in mouse embryo fibroblasts (MEFs) and several human cancer cell lines. Mechanistically, activating transcription factor 4 (ATF4) may bind to the promoter region of MTHFD2 and directly regulate its expression. In response to cell growth signals, mTORC1 may activate ATF4, which then promotes the expression of MTHFD2 and facilitates the production of formyl units required for *de novo* purine synthesis.

MYC is a master regulator of cell growth and proliferation. It participates in the regulation of cell cycle progression, genetic instability, apoptosis, and metabolism (30). By analyzing publicly available ChIP-Seq data and ChIP-qPCR assay, Pikman et al. (25) demonstrated that MYC could bind to the promoter region of MTHFD2 DNA in AML. Ju et al. (3) also showed that c-Myc could transcriptionally upregulate MTHFD2 expression in CRC through Kras associated pathway, including the PI3K/Akt and ERK pathways. The association between Kras and MTHFD2 gene expression has also been reported by Moran et al. (31) in non-small cell lung cancer (NSCLC).

Li et al. (32) reported that Ad4-binding protein/steroidogenic factor 1 (Ad4BP/SF-1) directly regulated MTHFD2 expression by binding to the CHIP-peak regions, thus affecting NADPH production in adrenocortical Y-1 cells. In Ewing sarcoma (EWS), the chimeric transcription factor EWS-FLI1 was the primary oncogenic driver that positively regulated the expression of MTHFD2 and MTHFD1L and impacted cellular redox status (33). SOX7 is a transcription factor and functions as a tumor suppressor. Zhang et al. (34) identified MTHFD2 as one of the essential target genes of SOX7 in breast cancer. They further demonstrated that SOX7-repressed MTHFD2 could contribute to SOX7-mediated tumor suppression. Besides, MTHFD2 was speculated as one of the regulatory targets of Nrf2, as MTHFD2 mRNA was decreased by Nrf2 knockdown in A549 lung cancer cells (35).

On the epigenetic level, MTHFD2 has been reported to be post-transcriptionally regulated by microRNAs (miRNAs). Transcriptome profiling in breast cancer cells identified MTHFD2 as a target gene of miR-9 that affected cell proliferation and induced apoptosis (36). In AML cells, miR-92a inhibited cell proliferation and promoted apoptosis by directly downregulating MTHFD2 (37). In glioma, miR-940 might disturb the 1C metabolic pathway and suppress tumor progression by regulating MTHFD2 (21). In CRC, miR-33a-5p inhibited the growth and migration of HCT116 and HT29 cells by targeting MTHFD2 (38).

Intriguingly, among the enzymes that involved in mitochondrial folate pathway, MTHFD2 was particularly responsive to extracellular stimuli. The intracellular protein level of MTHFD2 responded rapidly to mitogenic stimuli in several cancer cells, such as U251, HeLa, and HCT116 (10). The expression was repressed by the deprivation of growth signals (e.g., serum) within 24 h and could be rapidly re-induced within 4 h after serum re-stimulation. Enforced expression of MTHFD2 was sufficient to promote cancer cell proliferation in serum-deprived condition, indicating that its function might override the growth factor limitation (10, 39). This might, at least partially, contribute to the uncontrolled tumor growth even under nutrition limited environments and the poor efficacy of growth factor inhibitors (e.g., EGFR inhibitors) in some refractory cancers such as NSCLC and GBM. Thus, combination of MTHFD2 inhibitor and growth factor inhibitors might be a promising therapeutic strategy for EGFR inhibitor-resistant cancers.

## Intracellular Changes Caused by MTHFD2 Depletion

### Morphological Changes

Downregulating of MTHFD2 could affect cancer cell morphology and possibly impair the ability of migration and invasion. In AML cells, knockdown of MTHFD2 resulted in morphological shift including nuclear condensation and cytoplasmic ruffling (25). In breast cancer cells, MTHFD2 depletion caused a weaker and deformed vimentin network (24), indicating the impairment of cell motility.

### Increased Oxidative Stress

While the significance of folate metabolism has been recognized and attributed to the production of 1C units for nucleic acid synthesis, another crucial role of this pathway is the generation of NADPH, the important reducing power for redox homeostasis (40). Fan et al. (40) demonstrated that THF-mediated folate metabolism contributed as much as 40% of NADPH production in immortalized mouse kidney epithelial cells (iBMK). Knockdown of MTHFD2 led to a decreased NADPH/NADP^+^ ratio and increased reactive oxygen species level. In line with this study, Shin et al. (13) revealed that purified human MTHFD2 exhibited dual redox cofactor specificity and was able to utilize both NADP^+^ or NAD^+^ in rapidly proliferating cells. Additionally, Ju et al. (3) showed that MTHFD2 conferred redox homeostasis in CRC cells and promoted tumor growth and metastasis. Suppression of MTHFD2 disturbed NADPH production and redox homeostasis, rendering CRC cells more vulnerable to oxidative stress such as hypoxia. Histidine-induced filament formation required GCN2/ATF4/MTHFD2 axis-maintained redox homeostasis, and knockdown of MTHFD2 would affect the cytidine triphosphate (CTP) synthase filament formation due to redox imbalance (41). *Nmdmc*, the Drosophila homolog of MTHFD2, has been revealed as a longevity gene. Overexpression of *Nmdmc* extended Drosophila's lifespan, which might be associated with enhanced oxidative stress resistance. Decreased levels of mitochondrial ROS and Hsp22 and increased copy numbers of mitochondrial DNA were observed in *Nmdmc* upregulated Drosophila (42). It is not until recent years that the role of MTHFD2 in NADPH production and redox homeostasis has been recognized although the regulatory mechanism and clinical implication remain largely unknown.

### Metabolite Profile Alterations

Unsurprisingly, depletion of MTHFD2 would inhibit mitochondrial 1C metabolism and disturb purine synthesis. For instance, loss of MTHFD2 led to glycine auxotrophs (i.e., a reliance on exogenous glycine) in mammalian fibroblasts (43), breast cancer (44), and AML (25). In mammalian fibroblasts, knockout of *NMDMC(MTHFD2)* completely blocked 1-C unit generation in mitochondria, and the cytoplasmic folate pathways were insufficient to compensate for the optimal purine synthesis (43). Moreover, suppression of MTHFD2 in MCF-7 breast cancer cells caused prominent metabolic remodeling, such as greater vulnerability to exogenous folate depletion, enhanced glycolytic flux, and increased glutamine consumption (44). In addition to disturbing the serine-glycine conversion in mitochondria, MTHFD2 suppression may deplete the tricarboxylic acid (TCA) cycle intermediates and cholesterol esters and increase sphingomyelin and triglyceride levels (25). By tracing and measuring the isotope-labeled metabolites, Ben-Sahra et al. (29) demonstrated that depletion of MTHFD2 decreased *de novo* purine synthesis and was associated with reduced formate production.

MTHFD2-mediated purine synthetic metabolism has been demonstrated to be critical for stem-like cell properties and resistance to chemotherapy in lung cancer cells. Knockdown of MTHFD2 significantly reduced tumorigenesis and stem-like properties, probably due to insufficient purine nucleotide (28). The production of 5-aminoimidazole carboxamide ribonucleotide (AICAR), the final intermediate of purine synthesis pathway, is crucial to purine synthesis. MTHFD2 knockdown (or AICAR rescue) was found to reduce stem-like properties and restore sensitivity to gefitinib in gefitinib-resistant lung cancer cells. Overexpression of MTHFD2, on the other hand, conferred gefitinib resistance in gefitinib-sensitive cells. Taken together, this study suggested that MTHFD2-mediated 1C metabolism contributed to cancer stem-like properties and resistance to chemotherapy drugs through the consumption of AICAR.

In glioma, suppression of MTHFD2 through upregulation of miR-940 led to the disruption of intracellular 1C metabolism and exhibited anti-tumor effects (21). Interestingly, MTHFD2-dependent glycine synthesis has been reported as a prerequisite for angiogenesis in endothelial cells (45). Since abnormal angiogenesis is an important hallmark in GBM, targeting MTHFD2 may halt the progression of GBM by either slowing cancer cell proliferation or inhibiting abnormal angiogenesis, or both.

## The Role Beyond Enzymatic Function

Much less is known about the non-enzymatic activity of MTHFD2. Beside the canonical role of supporting purine synthesis and the newly-demonstrated role in redox defense. Recent studies conceptualized that MTHFD2 might profoundly regulate gene expression via affecting DNA replication, RNA translation, and epigenetic modification (Figure 2). Gustafsson Sheppard et al. (10) found that overexpression of MTHFD2 was sufficient to promote cancer cell proliferation independent of its dehydrogenase activity. They generated HCT-116 CRC cell lines expressing either the wild-type MTHFD2 protein or a mutant MTHFD2^Δ*NAD*^, which lacks the dehydrogenase activity due to a mutation in the NAD-binding site. Similar to wild-type MTHFD2, induction of MTHFD2^Δ*NAD*^ resulted in markedly increased cell proliferation, indicating that MTHFD2 protein could drive cell proliferation independent of its enzymatic function. They also found that MTHFD2 was co-localized with DNA replication sites in the nucleus, with a possible role in driving cancer cell proliferation. Koufaris et al. (39) investigated the possible non-enzymatic functions of MTHFD2 by identifying its interacting proteins, co-expression pattern and the knockdown transcriptional responses. By using co-immunoprecipitation (Co-IP) and mass spectrometry (MS), the authors identified that MTHFD2 may physically interact with a set of nuclear proteins involved in RNA metabolism and translation. Gene Ontology (GO) analysis of these proteins showed significant enrichment of RNA binding proteins, which were also frequently co-expressed with MTHFD2. A shared function between MTHFD2 and the interacting partners were supported by transcriptomics data.

The intriguing interactions between cell metabolism and gene expression have aroused extensive research attention in recent years. The reciprocally regulation of these two fundamental biological processes maintains homeostasis and regulates cell growth, survival, and differentiation (46). Cell metabolism has been established as an important regulator of eukaryotic gene expression, and there is a growing list of metabolic enzymes and metabolites with roles in the regulation of chromatin structure and transcription. Sdelci et al. (47) described the connection between 1C metabolism and gene transcriptional regulation. The folate metabolism enzyme MTHFD1 was bound to chromatin at distinct genomic loci and controlled gene expression in AML cells. The regulatory effect was dependent on the histone acetyl reader bromodomain-containing protein 4 (BRD-4), an important regulator chromatin structure and transcription. Other purine pathway enzymes, including SHMT and ADE2, have also been reported to interact with BRD4 bromodomains directly and may also transcriptionally regulate gene expression (48). More recently, MTHFD2 was reported to promoted metabolic reprogramming and tumor progression by forming a positive feedforward loop with HIF-2α. MTHFD2 promoted the methylation of HIF-2α mRNA and enhanced its translation, which in turn promoted the expression of MTHFD2 and aerobic glycolysis (49). This metabolic enzyme might therefore play an essential role in controlling RNA global N6-methyladenosine (m6A) methylation levels and linking RNA methylation status to the metabolic state.

A specific metabolic phenotype, elevated α-ketoglutarate to succinate ratio, could maintain pluripotency of embryonic stem cells through histone and DNA demethylation (50). Interestingly, MTHFD2 depletion could decrease the α-ketoglutarate to succinate ratio in AML cells and thus reduced their stem cell signatures, suggesting its potential role in epigenetic modulation.

## Development of MTHFD2 Inhibitors

Given the expression pattern of MTHFD2 and its knockdown effects in various cancers, there is a strong rationale for developing selective inhibitors targeting this enzyme for cancer therapy. The structure of MTHFD2 protein was first depicted in 2005 by Christensen et al. (51), its inhibitors has been discovered recently (Supplementary Table 2) As its enzyme activities require both the NAD and NADP cofactors and the substrate MTHF, one of the drug design strategies is to develop competitive inhibitors based on either the cofactors or the substrate. It is, however, worth noting that another two MTHFD enzymes (MTHFD1 and MTHFD2L) share both structural and functional similarities with MTHFD2. Tedeschi et al. (52) proposed a strategy to target MTHFD2 selectively but their modeling studies indicated that the conserved secondary and super-secondary structures (such as Rossman folds) in the three enzymes made it difficult to find competitive inhibitors with sufficiently high specificity. Notwithstanding, Nilsson et al. (15) demonstrated that MTHFD2 played predominant role in mitochondrial one-carbon pathway and should be the main targets. Inhibitors targeting multiple enzymes might result in a more complete inhibition.

The crystal structure of the first inhibitor of human MTHFD2, LY345899, was disclosed in 2017. Although this substrate-based competitive inhibitor has a lower affinity for MTHFD2 compared to MTHFD1, it showed potent suppressive effect on MTHFD2, with an IC50 value of 663 nmol/L (96 nmol/L on MTHFD1) (53). Notwithstanding, LY345899 treatment significantly inhibited CRC tumor growth in both cell lines and patient-derived xenograft (PDX) model (3). Following this, Kawai et al. (54) recently disclosed a novel isozyme-selective MTHFD2 inhibitor, DS44960156, with a tricyclic coumarin scaffold. It is characterized by several superior features, including remarkable selectivity (>18 fold) for MTHFD2 over MTHFD1, a low molecular weight (<400), and a good ligand efficiency (LE, a metric of binder). The upgraded compound DS18561882 showed a strong cell-based activity and a good oral pharmacokinetic profile which inhibited tumor growth in mouse xenograft breast cancer model upon oral administration (55). The anti-tumor efficacy of these selective MTHFD2 inhibitors in different cancers awaits further assessment. Moreover, the natural product carolacton, which was originally discovered as an antibacterial compound, was found to inhibit folate-dependent 1C metabolism by targeting FolD/MTHFD. Carolacton showed competitive inhibition on both the substrates and the cofactors, causing growth inhibition in different human cancer cell lines, such as HCT-116, KB-3.1, and KB- V.1 cells (56). Asai et al. (57) developed compounds that target THF and NAD pocket of MTHFD2, respectively, theie *in silico* study indicated high specificity and potential. But they have yet experimentally verify the efficacy of these compounds.

## Discussion

MTHFD2 has attracted increasing interests in cancer research due to its specific expression pattern and prognostic value. Agents that targeting this cancer cell specific molecular would minimize the adverse effects on normal cells. Several reports have demonstrated MTHFD2 as a critical player in cancer survival related to nucleotide synthesis, NADPH production, and redox defense. Depletion of MTHDF2 may abrogate malignant phenotypes, such as proliferation, migration, invasion, and metastasis. Cell line-derived xenograft and PDX-based studies further supported the anti-cancer effects of MTHFD2 knockdown *in vivo*. Currently, the studies are focus on breast cancer and gastrointestinal cancers, even its expression features and prognostic value have been demonstrate in a wider range of cancer types. It remains unknown whether inhibiting MTHFD2 in these cancers would significantly suppress tumor growth.

The expression of MTHFD2 can be regulated transcriptionally and post-transcriptionally or even by extracellular stimulation. The transcriptional factors that might regulate MTHFD2 expression have been identifying. But it remains elusive whether the high expression in cancer attributes to the MTHFD2-related genomic mutation or to the induction of other essential oncogenic drivers. How MTHFD2 might promote cancer progression remains undetermined. Most of the current studies indicated that oxidative stress defending would be an essential mechanism, but large is unknown especially from the non-enzymatic perspective. For instance, whether MTHFD2 is a DNA/RNA binding protein; what are the nucleotides it might bound; what are the genes it might regulated.

The inhibitory effects are predominantly carried out by RNA inference technique (siRNA and shRNA). Synthesized compounds has only been tested in breast cancer and CRC. The first synthetic MTHFD2 inhibitor LY345899 has shown potent anti-tumor activity in CRC while the research and development of inhibitors with higher affinity and selectivity is still ongoing. Its potential “moonlighting” functions in tumorigenesis, DNA replication, RNA metabolism, and epigenetic regulation render this molecule an intriguing research target. Genetical or pharmacological targeting MTHFD2 is a promising strategy in cancer therapy. Future studies may devote to investigate the anti-cancer role of MTHFD2 in a broad range of cancers; to clarify the regulatory factors and mechanisms attributing to the high expression of MTHFD2 in cancer cells; to explore the non-enzymatic functions of MTHFD2; to discover new compounds and test their efficacy in pre-clinical and clinical studies.

## Author Contributions

ZZ contributed to the literature research, manuscript draft, and figure/table design, GL provided critical revision of the manuscript as well as the final approval of the version to publish.

## Conflict of Interest

The authors declare that the research was conducted in the absence of any commercial or financial relationships that could be construed as a potential conflict of interest.
