# Supplementary material for: More Than a Metabolic Enzyme: MTHFD2 as a Novel Target for Anticancer Therapy?
Source: Front Oncol. 2020 Apr 28;10:658. doi: 10.3389/fonc.2020.00658 (PMC7199629; doi:10.3389/fonc.2020.00658)
Supplement: Supplementary file 1 [file Table_1.DOCX]

**Table 1.** The effects of MTHFD2 suppression in cancers.

| Cancer type | In vitro model | In vivo model | Tumour malignant phenotypes | Reference |
| --- | --- | --- | --- | --- |
| Breast cancer | HS578T  MCF7  MDA-MB-231 | - | Pro-death  Anti-proliferation  Anti-migration  Anti-invasion  Promote chemo-sensitivity | (1, 24, 35) |
| Acute myeloid leukaemia | U937  MOLM-14  THP-1  Primary cells | Cell-based xenograft | Anti-proliferation  Induce differentiation  Impair colony formation | (25) |
| Hepatocellular carcinoma | HepG2  Huh7 | - | Anti-migration  Anti-invasion  Suppress Epithelial–mesenchymal transition | (5) |
| Renal cell carcinoma | 786-O  CAKI-1 | Cell-based xenograft | Anti-proliferation  Anti-migration  Anti-invasion  Anti-tumour growth | (4, 48) |
| Colorectal cancer | HCT116  SW620  Lovo  CACO-2  RKO  SW-480 | Cell-based xenograft  PDX | Pro-apoptosis  Anti-tumour growth  Anti-metastasis  Anti-proliferation  Anti-cell migration  Inhibit G0/G1-S transition | (1, 3, 26, 56) |
| Lung cancer | H322  Adeno14-3 | Cell-based xenograft | Anti-proliferation  Reduce stem-like properties  Promote chemo-sensitivity | (27) |
| Glioma | U251 | - | Pro-death  Anti-proliferation | (1) |
| Ovarian cancer | OVCAR8 | - | Anti-proliferation | (1) |
| Melanoma | LOX IMVI | - | Anti-proliferation | (1) |
