# Supplementary material for: More Than a Metabolic Enzyme: MTHFD2 as a Novel Target for Anticancer Therapy?
Source: Front Oncol. 2020 Apr 28;10:658. doi: 10.3389/fonc.2020.00658 (PMC7199629; doi:10.3389/fonc.2020.00658)
Supplement: Supplementary file 2 [file Table_2.DOCX]

**Table 2.** The MTHFD2 inhibitors.

|  | Target(s) | Experimentally verify | Reference |
| --- | --- | --- | --- |
| LY345899 | MTHFD1/MTHFD2 | *In vitro* and *in vivo*:  colorectal cancer | (3, 52, 53) |
| Carolacton | MTHFD1/MTHFD2 | *In vitro*:  colon cancer  adenocarcinoma | (56) |
| MTHFD2 Inhibitor for THF pocket (MIT) | high specificity for MTHFD2 | - | (57) |
| MTHFD2 Inhibitor for NAD pocket (MIN) | high specificity for MTHFD2 | - | (57) |
| DS44960156 | high MTHFD2 affinity | - | (54) |
| DS18561882 | high MTHFD2 affinity | *In vitro* and *in vivo*:  breast cancer | (55) |
